# Risk of Dysglycemia in Pregnancy amongst Kenyan Women with HIV Infection: A Nested Case-Control Analysis from the STRiDE Study

**DOI:** 10.1155/2021/8830048

**Published:** 2021-04-05

**Authors:** Sonak D. Pastakia, Wycliffe K. Kosgei, Astrid Christoffersen-Deb, Benson Kiragu, John N. Hector, Gertrude Anusu, Ponnusamy Saravanan

**Affiliations:** ^1^Purdue University College of Pharmacy, Center for Health Equity and Innovation, 640 Eskenazi Ave, Indianapolis, IN 46202, USA; ^2^Academic Model Providing Access to Healthcare, Eldoret, Kenya; ^3^Moi Teaching and Referral Hospital, AMPATH Plus-RMNCAH/FP, Kenya; ^4^Department of Obstetrics and Gynaecology, University of British Columbia, Canada; ^5^Department of Obstetrics and Gynaecology, University of Toronto, Canada; ^6^Division of Health Sciences, Warwick Medical School, University of Warwick, UK; ^7^Academic Department of Diabetes & Endocrinology, George Eliot Hospital NHS Trust, Nuneaton, UK

## Abstract

**Introduction:**

Gestational diabetes is a common complication, whose incidence is growing globally. There is a pressing need to obtain more data on GDM in low- and middle-income countries, especially amongst high-risk populations, as most of the data on GDM comes from high-income countries. With the growing awareness of the role HIV plays in the progression of noncommunicable diseases and the disproportionate HIV burden African countries like Kenya face, investigating the potential role HIV plays in increasing dysglycemia amongst pregnant women with HIV is an important area of study.

**Methods:**

The STRiDE study is one of the largest ever conducted studies of GDM in Kenya. This study enrolled pregnant women aged between 16 and 50 who were receiving care from public and private sector facilities in Eldoret, Kenya. Within this study, women received venous testing for glycosylated hemoglobin (HbA1c) and fasting glucose between 8- and 20-week gestational age. At their 24-32-week visit, they received a venous 75 g oral glucose tolerance test (OGTT). Because of the pressing need to assess the burden of GDM within the population of pregnant women with HIV, a nested case-control study design was used. Pregnant women with HIV within the larger STRiDE cohort were matched to non-HIV-infected women within the STRiDE cohort at a 1 : 3 ratio based on body mass index, parity, family history of GDM, gestational age, and family history of hypertension. The measurements of glucose from the initial visit (fasting glucose and HbA1c) and follow-up visit (OGTT) were compared between the two groups of HIV+ cases and matched HIV- controls.

**Results:**

A total of 83 pregnant women with HIV were well matched to 249 non-HIV-infected women from the STRiDE cohort with marital status being the only characteristic that was statistically significantly different between the two groups. Statistically significant differences were not observed in the proportion of women who developed GDM, the fasting glucose values, the HbA1c, or OGTT measurements between the two groups. *Discussion*. Significant associations were not seen between the different measures of glycemic status between pregnant women with and without HIV. While significant differences were not seen in this cohort, additional investigation is needed to better describe the association of dysglycemia with HIV, especially in Kenyan populations with a higher prevalence of GDM.

## 1. Introduction

Gestational diabetes mellitus (GDM) is a common complication during pregnancy which can lead to harmful effects on both the mother and fetus. While this is a problem faced by women all over the world, the majority of research on GDM comes from high-income settings with limited research coming from low- and middle-income countries (LMIC). In most LMIC settings, especially those in Africa, there has been a much needed focus on the impact of chronic communicable illnesses like HIV on pregnancy while noncommunicable chronic illnesses like diabetes have received minimal funding from development agencies [1]. This focus has led to the introduction of large-scale HIV programs which have helped to expand access to highly active antiretroviral therapy (HAART) to millions of people across Africa [2]. Unfortunately, there are several noteworthy metabolic side effects which are related to infection with HIV and the medications used to treat it. While several studies from high-income countries have highlighted the potential for HIV/treatment to increase the risk of diabetes, there has only been limited investigation of the role HIV/treatment plays in influencing dysglycemia in pregnancy [3]. In a recent meta-analysis, HIV was not found to play a significant role in increasing the prevalence of GDM; however, the authors cautioned that additional prospective studies were needed to better analyze this dynamic [4]. Despite the disproportionately higher burden Africa faces from HIV, there is even less information on the role HIV plays in dysglycemia amongst pregnant women in this region. One of the few studies by Jao et al. in Cameroon found that HIV infection was not associated with GDM. However, in a subgroup analysis of the participants on antiretroviral therapy (*n* = 94 out of *N* = 166 people living with HIV (PLWH)), the investigators found that HIV treatment significantly increased the risk of developing GDM [5]. Therefore, more evidence is needed to better understand the dynamic between HIV and GDM and to potentially minimize the risks to pregnant women and their fetuses.

We explored the risk of GDM in PLWH compared to a matched cohort of non-HIV-infected pregnant women, in a nested case-control study within a large prospective early pregnancy cohort study. This cohort study (Stratification of Risk of Diabetes in Early Pregnancy (STRiDE) study) is one of the largest studies of women in early pregnancy to ever be carried out in Africa with the goal of understanding the role of ethnicity-specific risk factors and novel biomarkers for GDM.

## 2. Participants and Methods

The Kenyan site for the STRiDE study recruited patients from public and private sector settings in and around Eldoret. Most of the patients came from Eldoret, which is one of the fastest growing towns in Kenya with a population of ~475,000 [6]. The primary economic activity in this semiurban area revolves around agricultural activities typically involving farming of maize.

The overarching STRiDE study enrolled pregnant women aged between 16 and 50 years of age who presented early for antenatal care between 8 and 20 week gestational age. The study enrollment was between April 2015 and May 2019. Patients with a known history of type 1 or 2 diabetes, severe anemia (Hb < 8 mg/dL), sickle cell trait/disease, women on metformin, or any other serious illness were excluded from the study. Patients were recruited from the antenatal clinics of the different facilities included in the study. Upon providing informed consent and enrolling in the study, women were expected to complete three study visits during their pregnancy where different measures of glycemic control were assessed as described below: visit 1, initial visit between 8 and 20 weeks (HbA1c, fasting or random venous glucose (Cobas Analyzer, Roche Diagnostics, Basel, Switzerland)), and fasting or random capillary glucose (Optium H capillary glucose test, Abbott Diabetes Care, IL, USA)); visit 2, standard GDM screening between 24- and 32-week gestational age (75 g oral glucose tolerance test (OGTT), fasting venous glucose, one-hour post-75 g glucose load venous glucose, and two-hour post-75 g glucose load venous glucose); and visit 3, pregnancy outcome data assessment and routine glucose assessment during labor as clinically necessary for those with GDM. Patients were confirmed to have GDM based on the results of the 75 g OGTT completed during visit 2. A diagnosis of GDM was based on the International Association of Diabetes Study Group (IADPSG) criteria (one or more fasting, 1 h, or 2 h plasma glucose concentrations equal to or greater than threshold values of 5.1, 10.0, or 8.5 mmol/L, respectively) [7]. Within this analysis, only venous glucose levels were compared as they are the recommended testing approach for diabetes [7]. At each of these visits, a standardized questionnaire assessing demographic and clinical characteristics was administered with the information subsequently entered into an electronic database to facilitate future analysis. After providing informed consent at the first visit, a detailed history was taken including an assessment of their HIV/AIDS status as recommended by the National AIDS and Sexually Transmitted Infections Control Programme (NASCOP), body mass index (BMI), blood pressure utilizing an automatic Omron BP cuff (twice at 5-minute intervals), first-degree family history of diabetes and hypertension (parents, siblings, and number affected), personal history of polycystic ovarian syndrome (PCOS), educational attainment, socioeconomic status from the Demographic and Health Survey questionnaire (household income, disposable income, etc.), previous obstetric complications, and delivery history (type, birth weights and sex of previous children) [8]. Additional details regarding the STRIDE study have been described previously [9].

For this nested case-control study, all of the PLWH were identified from public sector facilities and were receiving care according to the NASCOP guidelines of Kenya [10]. The majority of the study patients 92.4% (3658) were recruited from different levels of the public sector health system which include Moi Teaching and Referral Hospital (MTRH), the second largest referral hospital in Kenya, Uasin Gishu District Hospital, and Langas Health Center. In addition to the public sector, a small number of patients 7.6% (301) were recruited from private sector facilities in Eldoret (Memorial, Reale, and Mediheal hospitals).

The HIV status of pregnant enrollees was the only HIV-related parameter collected for participants. During the duration of the study, the primary first-line antiretroviral regimen for women of child-bearing age in Kenya was an efavirenz-based regimen combined with a nucleos(t)ide transcriptase inhibitor (NRTI) backbone which typically included lamivudine and tenofovir. Second-line regimens included a protease-inhibitor- (PI-) based regimen combined with two NRTIs. Based on internal data from the public sector facilities where HIV patients were recruited for the STRiDE study, <5% of HIV patients were on PI-based second-line regimens in accordance with the national guidelines [10].

Principal Component Analysis (PCA) was used in the construction of the socioeconomic status (SES) scale. The SES scale assessed the pooled resources available to a person, family, or household. This included the standard measures of income per month, size of household, source of drinking water, type of toilet facility being used, and highest level of education.

Institutional Review Board approval was received for all study activities from the Moi University Investigational Review and Ethics Committee, University of Toronto Review and Ethics Board, and the Indiana University/Purdue University Indianapolis Institutional Review Board.

### 2.1. Statistical Analysis

The proposed nested case-control study was a secondary analysis of the main STRiDE study. The PLWH were matched with non-HIV-infected women from the STRiDE cohort at a ratio of 1 : 3 using MAHAPICK, a STATA® (College Station, TX) software module that seeks matching “control” observations for a set of “treated” observations. A 1 : 3 match was selected based on previously recommended statistical approaches for nested case-control studies which highlight that little power is added when adding more than 3 controls for each case [11, 12]. PLWH were matched based on the variables collected as part of the study which are known to potentially modulate the risk of dysglycemia. These included age, BMI, parity, family history of GDM, gestational age, and family history of hypertension.

Descriptive statistics were used to summarize the demographic characteristics of the population by calculating the medians and interquartile ranges for the relevant parameters.

The Wilcoxon rank-sum test was used to compare continuous variables and the chi-square test (*χ*^2^), or Fischer's exact test was used to analyze categorical variables to facilitate comparisons between the two groups. Continuous data were presented as mean ± standard deviation (SD) or medians (interquartile ranges) whereas categorical data was presented as percentages (numbers).

STATA SE 16 (College Station, TX, USA) statistical software was used in data cleaning and analysis, and a *p* value < 0.05 was considered statistically significant.

## 3. Results

**Figure 1:**
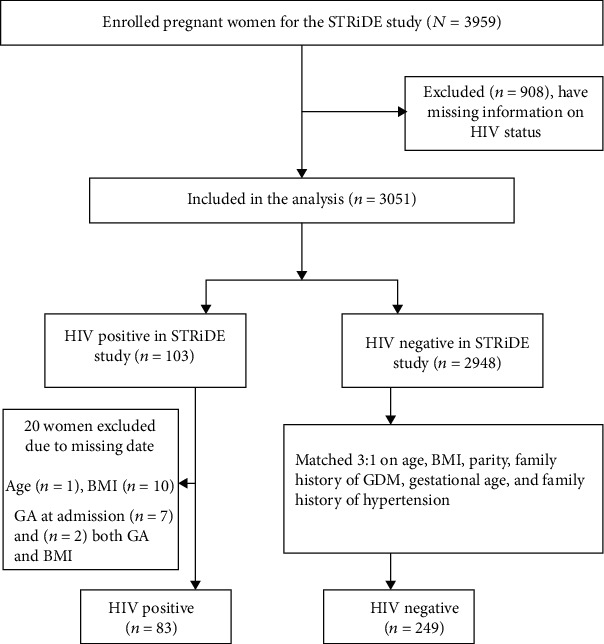
Flow chart for identification and analysis of patients.

PLWH were identified from the overarching STRiDE cohort of 3959 women. As seen in Figure 1, a total of 103 women living with HIV were identified with 20 women being excluded as they were missing key pieces of data required to appropriately match participants. The remaining 83 PLWH were matched with 249 non-HIV-infected women providing a total sample size of 332.

Baseline characteristics were well matched between HIV-negative participants and PLWH (Table 1).

The majority of women included in this analysis were between 25 and 35 years of age, were married, multiparous, and had normal BMI (20-24.9). While known hypertension was rare, a significant proportion had prior family history of GDM, diabetes, or hypertension. The tribal ethnicities of the participants were also well matched with majority of women coming from the Kalenjin ethnicity, representative of the population in this region of Kenya. The only significant difference between the two groups was the marital status of participants with PLWH being less likely to be married but more likely to be identified for enrollment at their first ANC visit.

**Figure 2:**
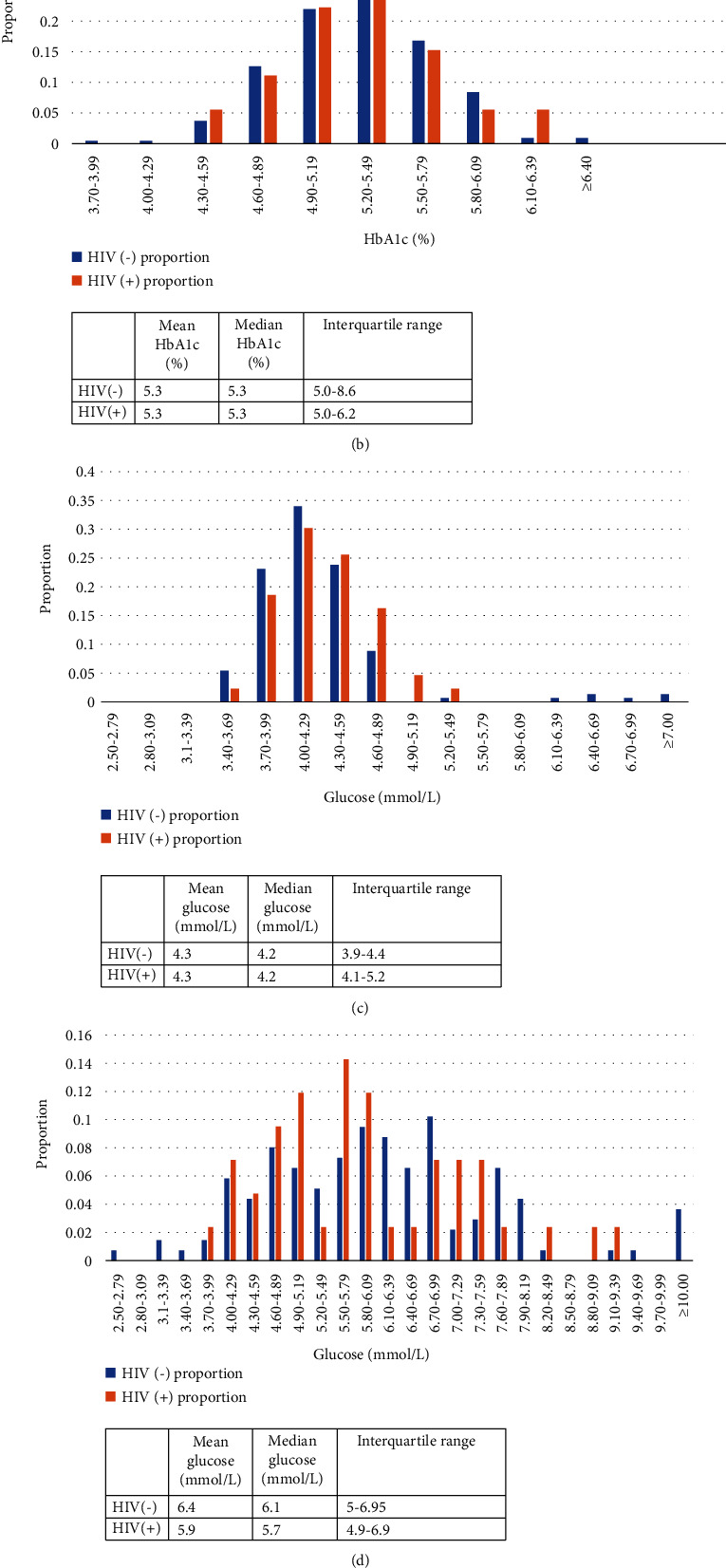
(a) Venous glucose values amongst those living with HIV vs. those without during initial visit prior to 18 weeks. (b) Venous HbA1c values amongst those living with HIV vs. those without during their 24-32-week visit. (c) Venous fasting glucose values amongst those living with HIV vs. those without during their 24-32-week visit. (d) Venous glucose values 60 minutes after 75 g glucose load amongst those living with HIV vs. those without during their 24-32-week visit. (e) Venous glucose values 120 minutes after 75 g glucose load amongst those living with HIV vs. those without during their 24-32-week visit.

Of the 332 women, 295 attended the blood sampling visit at baseline. The histogram of the glucose measurements in all stages of pregnancy in both groups is shown in Figures 2(a)–2(e).

One hundred and ninety women attended the OGTT visit. The characteristics of women who attended and did not attend were similar except for a statistically significant difference in BMI (Table 2).

The mean (±SD) gestational age and glucose measurements at OGTT were similar between the two groups (Table 3).

No statistically significant difference was observed in the proportion of pregnant women who developed GDM. Within the main analysis of differences in glycemic status between the two cohorts seen in Table 2, statistically significant differences were not seen. The different measures of glucose between the two cohorts were similar across all measurements. This included the median venous glucose taken at the initial visit, 4.3 mmol/L (IQR 4.0-4.5) for HIV-negative participants vs. 4.5 mmol/L (IQR 4.2-5.0) (*p* = 0.067) for PWLWH, median venous HbA1c at the initial visit (5.3% [IQR 5.0-5.5] for PWLWH vs. 5.3 [IQR 5.0-5.5] for HIV-negative participants, *p* = 0.72), and all parameters of the OGTT. Furthermore, differences in GDM positivity were not seen with 3.6% of HIV-negative participants vs. 2.4% (*p* = 0.595) of PWLWH being confirmed to be GDM positive.

As seen in Tables 4, 71.8% of the HIV-negative participants versus 45.7% of the PLWH had evaluable obstetric outcomes. For the populations with data available, significant differences were not seen in labor, pregnancy, or birth outcomes. Trends were seen in the birthweight as PLWH trended towards having lower birthweight babies and fewer large birthweight babies.

## 4. Discussion

In this study comparing a cohort of PLWH and a matched cohort of HIV-uninfected pregnant women, no significant associations between different measures of glycemic status in pregnancy and HIV positivity were found. Furthermore, this analysis highlights the relatively lower prevalence (2.4%) of GDM amongst PLWH in this semiurban Kenyan population compared to other PLWH populations where recent estimates from meta-analyses have found a prevalence of 7.1% in Asia, 5.8% in Europe, and 3.6% in the United States of America [14].

This observation provides further support for utilizing a risk-stratified screening approach to test only those with an elevated risk as proposed by investigators in Tanzania and under investigation by the broader STRiDE study [15].

This study is unique as it is one of the largest studies to compare multiple measures of dysglycemia amongst a cohort of PLWH who are matched to a similar cohort of non-HIV-infected women from the same setting. While most studies provide only an estimate of the prevalence of GDM in an HIV-infected population, without a comparator, this study design is uniquely able to elucidate the role of HIV in driving GDM by using a nested case-control methodology [14].

The study was limited by the relatively small sample size of PLWH who were enrolled within the broader STRiDE study and low prevalence of dysglycemia and GDM. The study was also limited by the lack of additional details on the management of HIV and the obstetric outcomes of those patients as that information was not available for a large proportion (47%) of the population. Data on their CD4+ count, viral load, and their antiretroviral regimen was not collected as part of the STRiDE dataset. This information, especially details of the type of antiretrovirals, would have been useful to include within this assessment as several studies have shown that PI use is associated with a significantly higher risk of developing diabetes mellitus [4, 16, 17]. A recent meta-analysis also found a trend towards a higher prevalence of GDM amongst PLWH who were on PI-based regimens [14]. While this is a noteworthy limitation of this study, the impact was likely to be minimal as very few participants in this setting are on PI-based antiretrovirals as <5% of the overarching HIV populations receiving care at facilities receive this regimen within this region. Furthermore, other HIV medications known to increase the risk of diabetes, such as stavudine, were being phased out of HIV care in Kenya during the period of enrollment for the study so it is unlikely many women were on these medications [3]. The findings from this study suggest that HIV itself plays a negligible role in the development of dysglycemia amongst the population seeking care in this semiurban region in Kenya. However, as seen within Figures 1(a)–1(d), the distribution of glucose measurements skewed towards being higher for PLWH; however, significant differences were not seen within the limited sample size available for this evaluation. While HIV infection does not appear to play a role in driving dysglycemia in this cohort, the findings are limited specifically to this cohort as populations and regions with a higher prevalence of diabetes/GDM might observe a larger modulating effect from HIV infection.

Furthermore, this investigation tried to control for the expected modulators of dysglycemia between PLWH and non-HIV-infected individuals through a case-control approach as opposed to the gold standard approach of a randomized control trial which would control for both known and unknown confounders. While marital status was the only characteristic which was significantly different at baseline, it is possible that additional socioeconomic factors which were not matched between the two cohorts may have obscured the assessment as HIV tends to disproportionately affect lower socioeconomic populations in this region.

While studies from other populations have found an increased risk of GDM amongst PLWH, the lack of an association within this population highlights the need for additional, large, setting-specific, randomized control trials, which assess the many different risk factors which impact the risk of dysglycemia. It is also possible that differences might become more apparent as the prevalence of dysglycemia continues to rise in the general population as semiurban settings like Eldoret continue to shift towards a more sedentary lifestyle [13, 18].

## Figures and Tables

**Table 1:** Baseline characteristics.

	All (*N* = 332) (%) (*N*)	HIV negative (249) (%) (*n*)	HIV positive (83) (%) (*n*)	*p* value
Age, median (IQR) in years	30 (26-34)	30 (26-34)	30 (25-35)	0.619
Age groupings				
18-24.9	21.4% (71)	21.3% (53)	21.7% (13)	0.943
25-34.9	54.5% (181)	55.0% (137)	53.0% (44)	
>35	24.1% (80)	23.7% (59)	25.3% (21)	
Marital status				
Currently married	86.5% (287)	89.2% (222)	78.3% (65)	0.032
Widowed	0.6% (2)	0.4% (1)	1.2% (1)	
Single/never married	6.6% (22)	4.4% (11)	13.3% (11)	
In a relationship/unmarried	5.7% (19)	5.2% (14)	7.2% (6)	
Missing	0.6% (2)	0.8% (2)	0	
Gestational age at enrollment (weeks), median (IQR)	15 (13-18)	15 (13-18)	15 (13-18)	0.972
Parity				
Nulliparous	16.9% (56)	16.9% (42)	16.9% (15)	0.983
1-3	69.3% (230)	69.5% (173)	68.7% (57)	
>3	13.9% (46)	13.7% (34)	14.5% (12)	
Previous miscarriages				
None	77.7% (258)	79.1% (197)	73.5% (61)	0.565
One or more miscarriage	20.2% (67)	18.9% (47)	24.1% (20)	
Missing	2.1% (7)	2.0% (5)	2.4% (2)	
BMI (kg/m^2^), median (IQR)	25(22-28)	25(22-28)	25(22-29)	0.776
BMI				
Underweight (<20)	9.3% (31)	8.4% (21)	12.1% (10)	0.537
Normal (20-26)	49.7% (165)	49.4% (123)	50.6% (42)	
Overweight (≥26)	41.0% (136)	42.2% (105)	37.4% (31)	
Family history of GDM	4.8% (16)	4.8% (12)	4.8% (4)	0.599
Family history of diabetes	20.0% (66)	19.3% (48)	21.7% (13)	0.634
Family history of hypertension	34.9% (116)	34.9% (87)	34.9% (29)	1.000
Elevated blood pressure	0.6% (2)	0.4% (1)	1.2% (1)	0.413
Ethnicity				
Kalenjin	34.3% (114)	37.0% (92)	26.5% (22)	0.179
Luhya	24.4% (81)	24.5% (61)	24.1% (20)	
Kikuyu	15.4% (51)	15.3% (38)	15.7% (13)	
Luo	7.83% (26)	6.02% (15)	13.3% (11)	
Others	7.23% (24)	8.03% (20)	4.8% (4)	
Unknown	5.42% (18)	4.42% (11)	8.4% (7)	
Kisii	5.12% (17)	4.42% (11)	7.2% (6)	
Missing	0.3% (1)	0.4% (1)		
Economic status				
Low	35.5% (118)	34.1% (85)	40.0% (33)	0.296
Medium	32.5% (108)	35.3% (88)	24.1% (20)	
High	8.1% (27)	8.0% (20)	8.4% (7)	
Missing	23.8% (79)	22.5% (56)	27.7% (23)	

**Table 2:** Baseline characteristics of participants who completed and did not complete the oral glucose tolerance test between 24 and 32 weeks.

	All (*N* = 332) (%) (*N*)	Attended OGTT‒no (142) (%) (*n*)	Attended OGTT‒yes (190) % (*n*)	*p* value
Age, median (IQR) in years	30 (26-34)	30 (26-34)	30 (26-35)	0.899
Age groupings				
18-24.9	21.4% (71)	25.4% (36)	18.4% (35)	0.310
25-34.9	54.5% (181)	52.1% (74)	56.3% (107)	
>35	24.1% (80)	22.5% (32)	25.3% (48)	
Marital status				
Currently married	86.5% (287)	87.3% (124)	85.8% (163)	0.560
Widowed	0.6% (2)	0.7% (1)	0.5% (1)	
Single/never married	6.6% (22)	7.6% (11)	5.8% (11)	
In a relationship/unmarried	5.7% (19)	3.5% (5)	7.4% (15)	
Missing	0.6% (2)	0.7% (1)	0.5% (1)	
Gestational age at enrollment (weeks), median (IQR)	15 (13-18)	15 (13-18)	16 (13-18)	0.104
Parity				
Nulliparous	16.9% (56)	14.1% (20)	19.0% (36)	0.246
1-3	69.3% (230)	69.0% (98)	69.5% (132)	
>3	13.9% (46)	16.9% (24)	11.6% (22)	
Previous miscarriages				
None	77.7% (258)	73.9% (105)	80.5% (153)	0.048
One or more miscarriage	20.2% (67)	25.4% (36)	16.3% (31)	
Missing	2.1% (7)	0.7% (1)	3.2% (6)	
BMI (kg/m^2^), median (IQR)	25 (22-28)	23 (21-27)	26 (23-30)	<0.001
BMI				
Underweight (<20)	9.3% (31)	14.1% (20)	5.8% (11)	<0.001
Normal (20-26)	49.7% (165)	57.0% (81)	44.2% (84)	
Overweight (≥26)	41.0% (136)	28.9% (41)	50.0% (95)	
Family history of GDM	4.8% (16)	4.2% (6)	5.3% (10)	0.798
Family history of diabetes	20.0% (66)	21.1% (30)	19.0% (36)	0.677
Family history of hypertension	34.9% (116)	31.7% (45)	37.4% (71)	0.297
Elevated blood pressure	0.6% (2)	1.4% (2)	0	0.182
HIV status	25.0% (83)	28.2% (40)	22.6% (43)	0.249
Ethnicity				
Kalenjin	34.3% (114)	35.2% (50)	33.7% (64)	0.775
Luhya	24.4% (81)	23.2% (33)	25.3% (48)	
Kikuyu	15.4% (51)	14.8% (21)	15.8% (30)	
Luo	7.8% (26)	7.8% (11)	7.9% (15)	
Others	7.2% (24)	9.9% (14)	5.3% (10)	
Unknown	5.4% (18)	4.2% (6)	6.3% (12)	
Kisii	5.1% (17)	4.9% (7)	5.3% (10)	
Missing	0.3% (1)	0	0.5% (1)	
Economic status				
Low	35.5% (118)	38.7% (55)	33.2% (63)	0.723
Medium	32.5% (108)	31.0% (44)	33.7% (64)	
High	8.1% (27)	8.5% (12)	7.9% (15)	
Missing	23.8% (79)	21.8% (31)	25.3% (48)	

**Table 3:** Glucose measurements at the oral glucose tolerance test visit between 24 and 32 weeks.

	All	HIV negative	HIV positive	*p* value
Venous glucose (mmol/L) at initial visit, median (IQR) [*n*]	4.4 (4.0-5.0) [295]	4.3 (4.0-4.5) [222]	4.5 (4.2-5.0) [73]	0.067

Venous HbA1c (%) at initial visit, median (IQR) [*n*]	5.3 (5.0-5.5) [288]	5.3 (5.0-5.5) [215]	5.3 (5.0-5.5) [73]	0.719

Plasma glucose (mmol/L) levels median (IQR) [*n*]				
Fasting	4.2 (4.0-4.5) [190]	4.2 (4.0-4.4) [147]	4.2 (4.0-4.6) [43]	0.171
60 min	5.9 (4.8-7.0) [190]	6.1 (4.7-7.0) [147]	5.7 (4.8-7.0) [43]	0.395
120 min	5.2 (4.4-6.2) [190]	5.2 (4.2-6.2) [147]	5.2 (4.4-5.8) [43]	0.698

GDM positivity				
Positive	3.3% [11]	3.6% [9]	2.4% [2]	0.595

**Table 4:** Birth and obstetric outcomes, amongst all *n* = 332 that have visit 4 data.

	All (*N* =176) % (*N*)	HIV negative (138) % (*n*)	HIV positive (38) % (*n*)	*p* value
Gestational age at delivery, median (IQR)	40 (39-40)	40 (38-40)	40 (39-40)	0.668
Preterm (20-36 weeks)	8.0% (15)	9.4% (14)	2.6% (1)	0.703
Term (37-41 weeks)	80.0% (140)	77.5% (107)	86.8% (33)	
Postterm (>42 weeks)	8.0% (15)	8.0% (11)	7.9% (3)	
Missing data	2.8% (5)	2.9% (4)	2.6% (1)	

Birthweight, median (IQR)	3.2 (1.5-4.5)	3.2 (1.5-4.5)	3.1 (1.7-4.2)	0.798
Low birth weight (<2500 g)	3.4% (6)	2.9% (4)	5.3% (2)	0.448
Normal birth weight (2500-4000 g)	83.5% (147)	81.9% (113)	89.5% (34)	
Large birth weight (>4000 g)	6.8% (12)	8.0% (11)	2.6% (1)	
Missing data	6.3% (11)	7.25% (10)	2.6% (1)	

Mode of delivery				
Vaginal delivery	78.4% (138)	76.1% (105)	86.8% (33)	0.560
Assisted vaginal delivery (vacuum)	0.6% (1)	0.72% (1)	0	
LTCS-elective	4.6% (8)	5.8% (8)	0	
LTCS-emergency	14.8% (26)	15.2% (21)	13.2% (5)	
Missing data	1.7% (3)	2.2% (3)	0	

Pregnancy outcome				
Live birth	93.2% (164)	92.0% (127)	97.4% (37)	0.916
Miscarriage	1.7% (3)	2.2% (3)	0	
Stillbirth	2.3% (4)	2.9% (4)	0	
Missing data	2.8% (5)	2.9% (4)	2.6% (1)	

LTCS: low transverse cesarean section.

## Data Availability

The data used to support the findings of this study are restricted by the Moi University Institutional Review and Ethics Board in order to protect patient privacy. Data are available from Ponnusamy Saravanan (p.saravanan@warwick.ac.uk+) for researchers who meet the criteria for access to confidential data as per University of Warwick's standard operating procedures 12 months after the publication of the main paper.
